# Supplementary material for: Resolving the full spectrum of human genome variation using Linked-Reads
Source: Genome Res. 2019 Apr;29(4):635–45. doi: 10.1101/gr.234443.118 (PMC6442396; doi:10.1101/gr.234443.118)
Supplement: Supplemental Material [file supp_gr.234443.118_Supplemental_longranger-2.2.zip › longranger-2.2.tar.bz2!/lr2.2-public/lib/stan/cmdstan/stan/doc/api/html/structstan_1_1gm_1_1name__vis.html]

Stan: stan::gm::name\_vis Struct Reference


|  |  |
| --- | --- |
|  | Stan  2.5.0  probability, sampling & optimization |


- Main Page
- Related Pages
- Namespaces
- Classes
- Files

- Class List
- Class Index
- Class Hierarchy
- Class Members

All Classes Namespaces Files Functions Variables Typedefs Enumerations Enumerator Friends Macros Pages

- stan
- gm
- name\_vis

Public Member Functions |
List of all members

stan::gm::name\_vis Struct Reference

`#include <ast.hpp>`

Inheritance diagram for stan::gm::name\_vis:

|  |  |
| --- | --- |
| Public Member Functions | |
|  | name\_vis () |
|  | |
| std::string | operator() (const nil &x) const |
|  | |
| std::string | operator() (const int\_var\_decl &x) const |
|  | |
| std::string | operator() (const double\_var\_decl &x) const |
|  | |
| std::string | operator() (const vector\_var\_decl &x) const |
|  | |
| std::string | operator() (const row\_vector\_var\_decl &x) const |
|  | |
| std::string | operator() (const matrix\_var\_decl &x) const |
|  | |
| std::string | operator() (const simplex\_var\_decl &x) const |
|  | |
| std::string | operator() (const unit\_vector\_var\_decl &x) const |
|  | |
| std::string | operator() (const ordered\_var\_decl &x) const |
|  | |
| std::string | operator() (const positive\_ordered\_var\_decl &x) const |
|  | |
| std::string | operator() (const cholesky\_factor\_var\_decl &x) const |
|  | |
| std::string | operator() (const cholesky\_corr\_var\_decl &x) const |
|  | |
| std::string | operator() (const cov\_matrix\_var\_decl &x) const |
|  | |
| std::string | operator() (const corr\_matrix\_var\_decl &x) const |
|  | |

## Detailed Description

Definition at line 571 of file ast.hpp.

## Constructor & Destructor Documentation

|  |  |  |  |  |
| --- | --- | --- | --- | --- |
| stan::gm::name\_vis::name\_vis | ( |  | ) |  |

Definition at line 1160 of file ast\_def.cpp.

## Member Function Documentation

|  |  |  |  |  |  |
| --- | --- | --- | --- | --- | --- |
| std::string stan::gm::name\_vis::operator() | ( | const nil & | *x* | ) | const |

Definition at line 1161 of file ast\_def.cpp.

|  |  |  |  |  |  |
| --- | --- | --- | --- | --- | --- |
| std::string stan::gm::name\_vis::operator() | ( | const int\_var\_decl & | *x* | ) | const |

Definition at line 1164 of file ast\_def.cpp.

|  |  |  |  |  |  |
| --- | --- | --- | --- | --- | --- |
| std::string stan::gm::name\_vis::operator() | ( | const double\_var\_decl & | *x* | ) | const |

Definition at line 1167 of file ast\_def.cpp.

|  |  |  |  |  |  |
| --- | --- | --- | --- | --- | --- |
| std::string stan::gm::name\_vis::operator() | ( | const vector\_var\_decl & | *x* | ) | const |

Definition at line 1170 of file ast\_def.cpp.

|  |  |  |  |  |  |
| --- | --- | --- | --- | --- | --- |
| std::string stan::gm::name\_vis::operator() | ( | const row\_vector\_var\_decl & | *x* | ) | const |

Definition at line 1173 of file ast\_def.cpp.

|  |  |  |  |  |  |
| --- | --- | --- | --- | --- | --- |
| std::string stan::gm::name\_vis::operator() | ( | const matrix\_var\_decl & | *x* | ) | const |

Definition at line 1176 of file ast\_def.cpp.

|  |  |  |  |  |  |
| --- | --- | --- | --- | --- | --- |
| std::string stan::gm::name\_vis::operator() | ( | const simplex\_var\_decl & | *x* | ) | const |

Definition at line 1182 of file ast\_def.cpp.

|  |  |  |  |  |  |
| --- | --- | --- | --- | --- | --- |
| std::string stan::gm::name\_vis::operator() | ( | const unit\_vector\_var\_decl & | *x* | ) | const |

Definition at line 1179 of file ast\_def.cpp.

|  |  |  |  |  |  |
| --- | --- | --- | --- | --- | --- |
| std::string stan::gm::name\_vis::operator() | ( | const ordered\_var\_decl & | *x* | ) | const |

Definition at line 1185 of file ast\_def.cpp.

|  |  |  |  |  |  |
| --- | --- | --- | --- | --- | --- |
| std::string stan::gm::name\_vis::operator() | ( | const positive\_ordered\_var\_decl & | *x* | ) | const |

Definition at line 1188 of file ast\_def.cpp.

|  |  |  |  |  |  |
| --- | --- | --- | --- | --- | --- |
| std::string stan::gm::name\_vis::operator() | ( | const cholesky\_factor\_var\_decl & | *x* | ) | const |

Definition at line 1191 of file ast\_def.cpp.

|  |  |  |  |  |  |
| --- | --- | --- | --- | --- | --- |
| std::string stan::gm::name\_vis::operator() | ( | const cholesky\_corr\_var\_decl & | *x* | ) | const |

Definition at line 1194 of file ast\_def.cpp.

|  |  |  |  |  |  |
| --- | --- | --- | --- | --- | --- |
| std::string stan::gm::name\_vis::operator() | ( | const cov\_matrix\_var\_decl & | *x* | ) | const |

Definition at line 1197 of file ast\_def.cpp.

|  |  |  |  |  |  |
| --- | --- | --- | --- | --- | --- |
| std::string stan::gm::name\_vis::operator() | ( | const corr\_matrix\_var\_decl & | *x* | ) | const |

Definition at line 1200 of file ast\_def.cpp.

---

The documentation for this struct was generated from the following files:

- src/stan/gm/ast.hpp
- src/stan/gm/ast\_def.cpp


---

[ Stan Home Page ]

*© 2011–2014,
Stan Development Team.*
